# Knockdown of LGR5 suppresses the proliferation of glioma cells *in vitro* and *in vivo*

**DOI:** 10.3892/or.2013.2826

**Published:** 2013-10-31

**Authors:** DONGLIANG WANG, JINGRU ZHOU, CUNGANG FAN, FENG JIAO, BO LIU, PENG SUN, JUNJIE MIAO, QINGJUN ZHANG

**Affiliations:** Department of Neurosurgery, Peking University People's Hospital, Beijing 100044, P.R. China

**Keywords:** glioma, LGR5, proliferation, orthotopic xenograft, siRNA

## Abstract

Leucine-rich repeat containing G protein-coupled receptor 5 (LGR5), one of the target genes of the Wnt signaling pathway, has recently been identified as a marker for brain cancer stem-like cells. However, the role of LGR5 in glioma is poorly understood. The aim of the present study was to investigate the relationship between LGR5 expression and pathological grade in glioma, and the impact of LGR5 on the proliferation of glioma cells *in vitro* and *in vivo*. Firstly, LGR5 expression was immunohistochemically evaluated in 54 resected gliomas of different pathologic grades, and its association with Ki-67 was evaluated. Subsequently, using western blotting and qRT-PCR, the expression of LGR5 was assessed in three glioma cell lines U87, U118 and U251. Moreover, the effects of LGR5 knockdown by siRNA on glioma cell proliferation, cell cycle, clone formation and tumorsphere formation *in vitro* and gliomagenesis *in vivo* were assessed. The results revealed that i) LGR5 was positively expressed in all glioma specimens and its expression increased with pathologic grade and Ki-67 expression; ii) LGR5 was highly expressed in three glioma cell lines and its expression was reduced significantly by siRNA; and iii) RNAi-mediated downregulation of endogenous LGR5 in U87 cells resulted in the suppression of cell proliferation, arrest of the cell cycle, and reduction in clone and tumorsphere formation *in vitro*. In addition, LGR5 depletion significantly inhibited tumor orthotopic xenograft growth in nude mice. These findings indicate that LGR5 plays a major role in gliomagenesis by promoting neoplastic cell proliferation, suggesting LGR5 as a molecular marker for pathology and a novel therapeutic target for malignant glioma.

## Introduction

Gliomas, the most common type of primary brain tumors in adults, are subcategorized by histopathologic evaluation and clinical criteria into four grades (I–IV), according to the current World Health Organization (WHO) guidelines (1). Despite attempts at treatment using surgical resection, radiation and chemotherapy with the alkylating agent temozolomide, the survival rates of patients with high-grade gliomas are less than 10% at 5 years (2). The extremely poor prognosis of these patients are due to the biological characteristics of glioma cells which include unrestricted proliferation and extensive invasion (3). Therefore, understanding the main molecular mechanisms of this malignancy is the key for the development of novel and effective therapeutic strategies for gliomas.

The leucine-rich repeat-containing G protein-coupled receptor 5 (LGR5) was initially identified as an orphan G protein-coupled receptor, containing a large extracellular domain with 17 leucine-rich repeats and a seven transmembrane domain (4). Recently, LGR5 is regarded as a somatic stem cell marker that plays key functional roles in normal development (5,6). Studies have demonstrated that LGR5 is highly expressed during embryonic development but is detected only in stem cells in postnatal tissues including the intestine, stomach, hair follicles and kidney (7–10).

Significantly, research has demonstrated that LGR5 plays a role in tumor progression likely due to mutational activation of the Wnt pathway (11). Accumulating evidence in human cancer cell lines has now confirmed that LGR5 promotes the growth and survival of ovarian carcinoma (12), basal cell carcinoma (13), colorectal and gastric carcinoma, esophageal adenocarcinoma (9,10,14,15) and liver carcinoma (16). Rcently, Nakata *et al*(17) demonstrated that expression of LGR5 is correlated with WHO grade in clinical samples of astrocytoma and that depletion of LGR5 induced apoptosis in brain cancer stem-like cells (CSCs). Thus, there is compelling evidence that LGR5 contributes to cancer initiation and progression. However, the exact mechanisms of LGR5 in glioma cells have not been elucidated.

In the present study, we confirmed the expression of LGR5 in human gliomas and its correlation with pathologic grade and proliferation. Moreover, we explored the role of LGR5 in the proliferation of U87 cells *in vitro* and *in vivo* by knockdown of LGR5 with RNA interference.

## Materials and methods

### Patients and specimens

Specimens from 54 patients with glioma who underwent surgical resection at the Department of Neurosurgery, Peking University People's Hospital between November of 2009 and May of 2012 were collected. Of the patients, 29 were male and 25 were female. Ages of the patients at the time of surgery ranged from 21 to 75 years [mean age ± standard deviation (SD), 45.8±14.5 years]. According to the revised WHO criteria for the central nervous system (18), tumors were categorized into grade I (n=5), grade II (n=13), grade III (n=13) and grade IV (n=23). All tumor tissues were obtained from the initial surgery, and none of the patients had been subjected to chemotherapy or radiation therapy prior to tumor excision. The histologic subtypes and pathologic grades of all glioma samples were confirmed by two pathologists independently. The present study was approved by the Institutional Review Board, and all participants provided written informed consent.

### Immunohistochemistry

All paraffin-embedded sections were deparaffinized followed by washing in xylenes and serial dilutions of ethanol. Endogenous peroxidase was blocked by 3% H_2_O_2_ for 12 min. After antigen retrieval, blocks for avidin and biotin and the Fc receptor were applied. The rabbit anti-LGR5 polyclonal antibody was used at a 1:150 dilution (Abcam, Cambridge, UK) or the rabbit anti-human Ki-67 polyclonal antibody was used at a 1:200 dilution (Santa Cruz Biotechnology, Santa Cruz, CA, USA) overnight at 4°C in a humidified chamber. The primary antibodies were then detected using the appropriate labeled streptavidin-biotin (LSAB) kit (Fuzhou Maixin Biotechnology, Fuzhou, China) according to the manufacturer's instructions. Immunolabeled sections were visualized with 3′,3′-diaminobenzidine tetrahydrochloride (DAB; Sigma, St. Louis, MO, USA) and counterstained with hematoxylin. As control, phosphate-buffered saline (PBS) was used instead of the primary antibody.

### Evaluation of the staining results

The staining results for immunohistochemistry were evaluated by two independent neuropathologists who were blinded to clinical information. Brown-yellow staining in the cytoplasm and/or membrane was considered positive for LGR5. Brown-yellow staining in the nucleus was positive for Ki-67. To measure the LGR5 immunoreactivity score (IRS) and proliferative index (PI), 10 high-power (x400) fields (~1,000 cells) were randomly chosen for quantification in the most strongly stained tumor area of each section. The LGR5 staining intensity (LGR5-SI), the percentage of LGR5-positive tumor cells (LGR5-PP), and the resulting LGR5 immunoreactivity score (LGR5-IRS) were evaluated by a modified method as previously described (19,20). Briefly, the immunoreactivity score (LGR5-IRS: negative, 0; weak, 1–3; moderate, 4–6; strong, 8–12) was determined by multiplication of the value for LGR5 staining intensity (LGR5-SI: 0, no staining; 1, weak staining; 2, moderate staining; 3, strong staining) and the value for the percentage of LGR5-positive tumor cells (LGR5-PP: 0, <1%; 1, 1–25%; 2, 26–50%; 3, 51–75%; 4, >75%). Due to the heterogeneous staining intensity of the tumor cells, the SI was determined according to the staining intensity noted in the majority of the cells. The percentage of Ki-67-positive cells was regarded as the PI of each tumor tissue sample, respectively.

### Cell culture

Human malignant glioma cell lines (U118, U87 and U251) and normal human astrocytes (1800) were obtained from the Cell Library of the Chinese Academy of Sciences (Shanghai, China). U118, U87 and U251 cells were cultured at 37°C in 5% CO_2_ in Dulbecco's modified Eagle's essential medium (DMEM) (Gibco, Carlsbad, CA, USA) supplemented with 10% fetal bovine serum (FBS; HyClone, Logan, UT, USA), 2 mM L-glutamine and 100 U/ml penicillin-streptomycin (Gibco). The normal astroctyes (1800) were cultured at 37°C in 5% CO_2_ in modified RPMI-1640 (HyClone) supplemented with 10% FBS, 2 mM L-glutamine and 100 U/ml penicillin-streptomycin (Gibco). The medium was changed every 3–4 days, and cultures were split using 0.25% trypsin. U87, U87-NC and U87-KD fluorescent (EGFP)-labeled cells were developed (Shanghai GeneChem Co, Ltd., Shanghai, China) and an *in vivo* optical imaging technique was used.

### Quantitative real-time polymerase chain reaction (qRT-PCR)

Total RNA was isolated from cells using the RNeasy Mini kit, including DNase treatment (Qiagen K.K., Tokyo, Japan). cDNA was synthesized using the PrimeScripts RT reagent kit (Perfect Real-Time; Takara Bio, Shiga, Japan) and qPCR was performed on a Thermal Cycler Dice Real-Time System using SYBR Premix Ex Taq™ (Perfect Real-Time). The primer sequences for qPCR were as follows: GAPDH forward, 5′-ATCATCCCTGCCTCTACTGG-3′ and GAPDH reverse, 5′-TTTCTAGACGGCAGGTCAGGT-3′; LGR5 forward, 5′-GAGGATCTGGTGAGCCTGAGAA-3′ and LGR5 reverse, 5′-CATAAGTGATGCTGGAGCTGGTAA-3′. GAPDH was used as a reference. Fold induction values were calculated using the 2^−ΔΔCt^ method. All experiments were performed in triplicate and repeated at least three times in separate experiments; representative data are shown.

### Western blotting

Cell lysates or the tissues dissolved in SDS sample buffer were separated by SDS-PAGE and transferred to nitrocellulose membranes. β-actin was used as a control. Membranes were probed for the LGR5 antibody (1:1,500; Abcam) or β-actin (1:2,000; Abcam) in Tris-buffered saline (TBS) containing 1% milk and 0.05% Tween-20 overnight at 4°C. The secondary antibody was horseradish peroxidase (HRP)-goat anti-rabbit or anti-mouse IgG (1:2,500; Cell Signaling) and incubation ws carried out for 1 h at room temperature. Blots were developed with Amersham ECL Western Blotting Detection reagent (GE Healthcare, Chalfont St. Giles, UK).

### siRNA

For knockdown of human LGR5, small hairpin RNA (shRNA) of the human LGR5 lentivirus gene transfer vector encoding green fluorescent protein (GFP) sequence was constructed by Shanghai GeneChem Co., Ltd. (Shanghai, China). The targeting sequence of the shRNA was 5′-GTCTGCAATCAGTTACCTA-3′. The recombinant lentivirus of small interference RNA targeting LGR5 (LGR5-RNAi-Lentivirus) was prepared and titered to 10^9^ TU/ml (transfection units/ml). A scrambled short-hairpin RNA (shRNA) was used as a negative control.

### MTT assay

An MTT assay was performed to detect the anti-proliferative effect of LGR5 RNA interference on U87 cells. U87 cells were seeded in 96-well plates at a density of 2×10^3^/well. After 24 h of incubation, cells were serum starved overnight. In another experiment, U87-NC-shRNA and U87-LGR5-shRNA cells were seeded in 96-well plates at a density of 2×10^3^/well and incubated for 24, 48, 72, 96 or 120 h. At each time-point, 20 ml of 5 mg/ml MTT (Sigma) solution was added to each well. After 4 h of incubation, the medium was removed from the wells by aspiration, and the formazan crystals were dissolved in 150 ml of dimethyl sulfoxide (DMSO; Sigma). Color intensity was measured at 490 nm with an enzyme linked immunosorbent assay plate reader (Bio-Rad Laboratories, Hercules, CA, USA). Cell growth curves were determined using the average absorbance at 490 nm from triplicate samples of three independent experiments.

### Cell cycle analysis

Cells were cultured in 25-ml flasks and incubated until they reached 60–70% confluence in DMEM containing 10% FBS. The cells were collected and washed twice with ice-cold PBS, and then fixed overnight with 70% ethanol at 4°C. Following incubation with 50 mg/ml RNase A at room temperature for 30 min, the cells were stained with 20 mg/ml propidium iodide (PI; Sigma) for an additional 30 min. DNA content and cell cycle distribution were analyzed by flow cytometry (FACSCalibur; Becton-Dickinson, San Jose, CA, USA) and the results were interpreted using ModFit and CellQuest software. All of the samples were assayed in triplicate.

### Plate colony formation assay

Cells (800 cells/plate) were cultured in 3 ml of DMEM supplemented with 10% FBS and 800 mg/ml G418 in a 6-well plate. After 2 weeks, colonies were rinsed with PBS, fixed with methanol for 5 min, and stained with Giemsa (Sigma) for 20 min. Clearly visible colonies (>50 mm in diameter) were counted as positive for growth.

### Tumorsphere formation assays

For tumorsphere formation, single-cell suspensions were suspended in Dulbecco's modified Eagle's medium/F12 (DMEM/F12; HyClone) supplemented with B-27 (1X, Gibco), 20 ng/ml epidermal growth factor (EGF; PeproTech Inc., Rocky Hill, NJ, USA) and 20 ng/ml basic fibroblast growth factor (bFGF; Peprotech) and then plated in 24-well ultra-low attachment plates (Corning Incorporated, Corning, NY, USA) at a concentration of 1,000 cells/well. Plates were analyzed 7–10 days later for tumorsphere formation, which was quantified using an inverted microscope (Olympus) at ×100 magnification.

### In vivo tumorigenicity assays

In an orthotopic model, 6-week-old BALB/C nude mice (Cancer Institute of the Chinese Academy of Medical Science) were randomly divided into three groups (three mice per group). All experiments were performed following approval of the Animal Studies Ethics Committee of the Peking University People's Hospital. A small burr hole, 2 mm in diameter was made (1 mm to the midline and 0.5 mm anterior to the bregma) using a microskull drill. A trochar packed with donor tissue was navigated to a depth of 2.5 mm via the skull hole. Cells (5×10^5^) (U87, Si-NC and Si-LGR5 cells) suspended in 5 μl PBS were slowly and smoothly injected into the subcortex of the mouse brain. The skull hole was sealed with bone wax and the scalp was sutured. Tumor growth in mice was detected by the Kodak In-Vivo FX Pro system (Kodak, Rochester, NY, USA). The tumor volume of the xenografts was detected every 5 days over the course of the study using fluorescence signaling.

Prior to the *in vivo* imaging, the mice were anesthetized with phenobarbital sodium. Fluorescence imaging was carried out with an excitation wavelength of 490 nm and emission wavelength of 535 nm. Exposure times ranged from 1 to 2 min. Mice were sacrificed and examined 5 weeks after the implantation. LGR5 was detected by immunohistochemistry.

### Statistical analysis

The experiments were performed in triplicate and repeated three times independently. SPSS 16.0 was used for all statistical analysis. Comparisons among all groups were performed using one-way analysis of variance (ANOVA). The t-test was used for comparison of differences between two groups. Correlation coefficients of LGR5 IRS with the PI were evaluated using Pearson correlation analysis. Values of P<0.05 were considered to indicate statistically significant results in all cases.

## Results

### Expression of LGR5 and its association with pathologic grade and proliferation index (PI) in glioma

**Figure 1:**
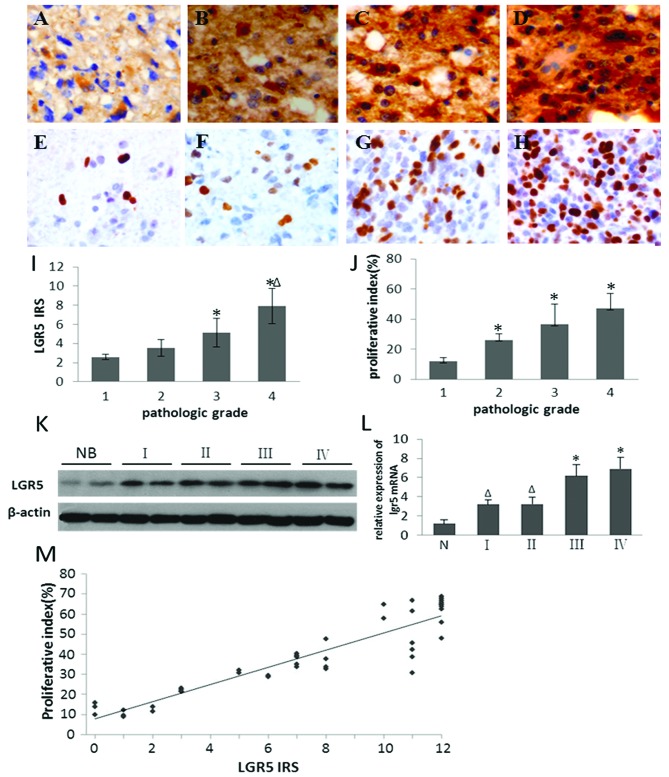
Expression of LGR5 is associated with pathologic grade and PI in glioma. (A–D) Immunohistochemical expression of LGR5 in glioma specimens of different grade. LGR5 immunoreactivity shows homogeneous brown-yellow staining in the cytoplasm of tumor cells; hematoxylin counterstain. (E–H) Immunohistochemical expression of Ki-67 in glioma specimens of different grade. Ki-67 immunoreactivity shows brown-yellow staining in the nucleus of tumor cells; hematoxylin counterstain. (A and E) Grade I; (B and F) grade II; (C and G) grade III; (D and H) grade IV. Original magnification, ×400. With increasing pathologic grade, the LGR5 IRS (I) and PI (J) in human gliomas were significantly increased (P<0.05; ^*^compared with grade I; ^Δ^compared with grade II). (K) Western blot analysis revealed a high level of expression of LGR5 in gliomas. β-actin was assessed as a loading control. Lanes NB, human normal brain tissue; lanes I, II, III, IV represent glioma grades, respectively. (L) qRT-PCR analysis revealed a high level of expression of LGR5 in gliomas. N, normal brain tissue; I, II, III, IV represent glioma grades, respectively. (P<0.05; ^*^compared with grade I; ^Δ^compared with N). (M) Scatterplots showing the correlation of LGR5 IRS with PI in human gliomas. A trend line is provided in each plot, which represents the ‘best fit’ as determined by simple linear regression. With increased LGR5 IRS, the PI was significantly increased (P<0.05).

In the present study, LGR5 protein was overexpressed in human glioma specimens. Positive tumor cells showed primarily cytoplasmic and/or membranous labeling under a light microscope. However, normal brain tissues had exceedingly weak immunoreactivity for this protein (data not shown). The LGR5 IRS was 4.60±2.57 for 54 cases of tumor specimens. The LGR5 IRS was positively and markedly correlated with increasing WHO grade (Fig. 1I; Table I). There were significant differences in LGR5 IRS between grade I and grade III (P<0.05), grade I and grade IV (P<0.005), and grade II and grade IV (P<0.05) gliomas, respectively. Representative images of LGR5 immunostaining are shown in Fig. 1A–D, and the related results are provided in Table I. The results of the western blot analysis and RT-PCR were coincident with that of immunohistochemistry. The results showed that mRNA and protein expression levels of LGR5 markedly increased with an increase in pathologic grade of the brain gliomas (P<0.05, Fig. 1K and L).

Pathologic grade has been linked to cell proliferation. Therefore, we sought to determine whether high expression of LGR5 might also be associated with proliferation. To address this we first compared LGR5 expression with proliferation. The proliferative index (PI) of tumor cells was evaluated by Ki-67 staining. The cell proliferation marker Ki-67 was expressed in all tumor specimens (Fig. 1E–H). With increasing pathologic grade of glioma, PI increased markedly (Fig. 1J; Table I). Moreover, the PI was positively correlated with LGR5 IRS (r=0.886, P<0.0001) (Fig. 1M).

### LGR5 is overexpressed in glioma cell lines, and LGR5 interference in U87 cells markedly reduces its expression

**Figure 2:**
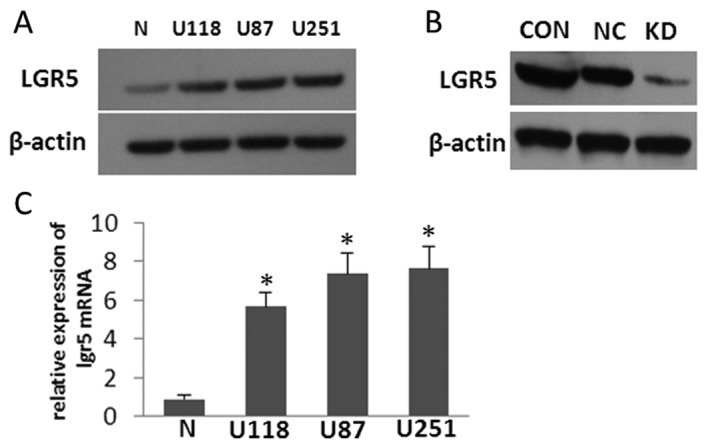
LGR5 is overexpressed in glioma cell lines, and LGR5 interference reduces its expression in U87 cells. (A) Protein levels in normal cultured primary astrocytes and in U118, U87 and U251 glioma cells as assessed by western blot analysis. (B) RNA interference reduced the expression of LGR5 in U87 cells as assessed by western blot analysis. Con, parental U87 cells; NC, negative control; KD, LGR5 knockdown cells. (C) mRNA levels in normal cultured primary astrocytes and in U118, U87 and U251 glioma cells as assessed by qRT-PCR analysis. N, normal cultured primary astrocytes.

The expression levels of LGR5 mRNA and protein in several high grade glioma-derived cell lines (U118, U87 and U251) cultured *in vitro* were compared to the expression levels in normal human cultured primary astrocytes by western blot analysis and qRT-PCR (Fig. 2A and C). The results showed that LGR5 was highly expressed in the three glioma cell lines when compared with that in the normal astrocytes; U87 cells were randomly used as an appropriate *in vitro* model for assessing LGR5 function in the subsequent experiments. To study the role of LGR5 in the malignant progression of glioma, we established a stably transfected U87 cell line expressing shRNA against LGR5. The protein level of LGR5 was confirmed to be dramatically downregulated in the U87-KD cells when compared to the parental U87 and U87-NC cells. In addition, there was no obvious difference in LGR5 expression between the control cell lines (Fig. 2B). These data indicate that transfection of LGR5 shRNA significantly and specifically inhibited the endogenous LGR5 expression in U87-KD human glioma cells.

### Knockdown of LGR5 inhibits U87 cell growth in vitro

**Figure 3:**
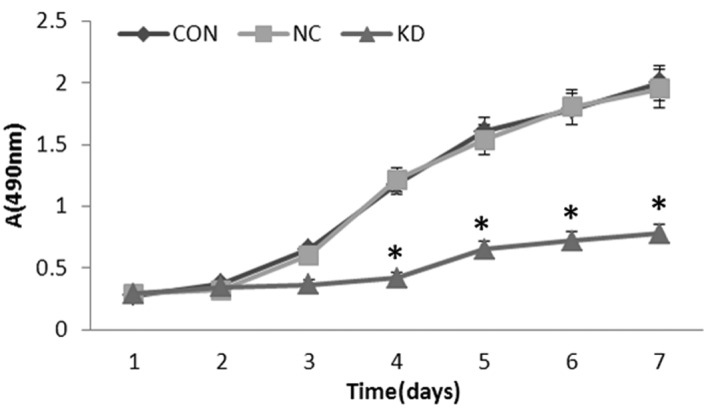
RNAi-mediated knockdown of LGR5 inhibits growth of U87 cells *in vitro*. Cell viability was measured using an MTT assay. Cell growth curves were determined by absorbance at 490 nm (^*^P<0.01 for U87-KD relative to each of the three control lines). Con, parental U87 cells; NC, negative control (shRNA-Ctr/U87); KD, LGR5 knockdown cells (shRNA-LGR5/U87).

To evaluate the effect of LGR5 on the growth of U87 cells, viability curves for U87, U87-NC and U87-KD cells were determined by MTT assay. As shown in Fig. 3, the growth of U87-KD cells was obviously inhibited when compared with that in the other groups, with this effect being most obvious from day 3 to day 7 (P<0.01). However, there were no significant differences in cell growth between the U87 and U87-NC cells (P>0.05). These results indicate that downregulation of LGR5 expression by RNAi markedly inhibited the growth of U87 cells.

### Knockdown of LGR5 inhibits cell cycle progression of U87 cells in vitro

**Figure 4:**
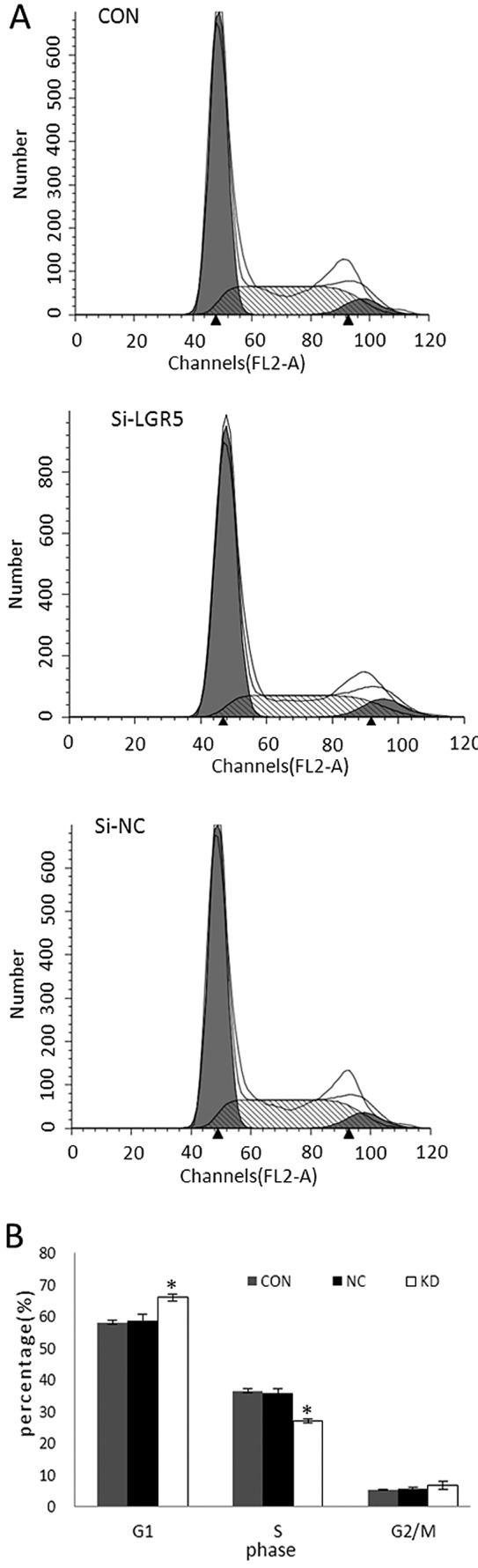
RNAi-mediated knockdown of LGR5 affects the cell cycle distribution of U87 cells *in vitro*. (A) Parental U87 cells (CON), shRNA-LGR5/U87 cells [Si-LGR5 (KD)] and shRNA-Ctr/U87 (Si-NC) cells were stained with propidium iodide (PI). The DNA content and cell cycle distribution were examined and analyzed by flow cytometry. (B) The histogram shows the percentages of cells in each cell cycle phase as determined by gating of the flow cytometry..

To investigate the effect of LGR5 knockdown on cell cycle progression, flow cytometry was performed to determine the cell cycle distribution (Fig. 4A). Compared with parental U87 and U87-NC cells, U87-KD cells accumulated in the G0/G1 phase (P<0.05), whereas the percentage of cells distributed in the S phase was decreased sharply (P<0.05). There was no obvious difference in cell cycle distribution between the parental U87 and U87-NC cells (P>0.05; Fig. 4B). These results suggest that reduction in LGR5 expression in U87 cells by RNAi delays cell cycle progression and decreases cell proliferation.

### Knockdown of LGR5 inhibits colony formation in vitro

**Figure 5:**
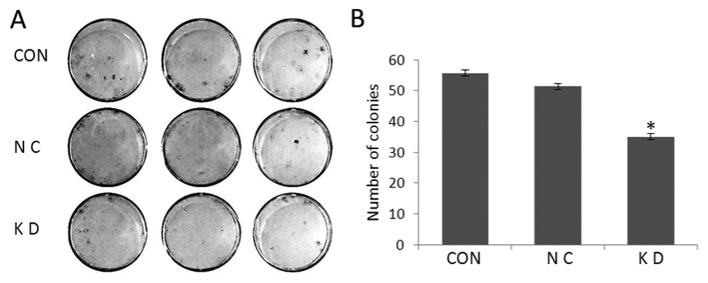
LGR5 knockdown suppresses plate colony formation. Equal numbers of parental U87, U87-NC and U87-KD cells were seeded onto 6-well plates. (A) The cells were fixed and stained with Giemsa after 14 days. (B) The number of colonies in U87-KD cells was significantly less than that in the U87-NC and non-transfected U87 cells (^*^P<0.05). CON, parental U87 cell; NC, negative control (shRNA-Ctr/U87); KD, LGR5 knockdown cells (shRNA-LGR5/U87).

To analyze the effect of LGR5 downregulation on the anchorage-dependent growth potential of U87 cells, plate colony formation assays were performed for parental U87, U87-NC and U87-KD cells (Fig. 5A). Compared to the control cells, the number and size of colonies for U87-KD cells were significantly decreased (P<0.05; Fig. 5B). In contrast, there were no obvious differences in colony-forming ability between the control cell lines (P>0.05). These data indicate that reduction in LGR5 expression decreased the colony-formation ability of U87 cells *in vitro*.

### Knockdown of LGR5 negatively regulates tumorsphere formation

**Figure 6:**
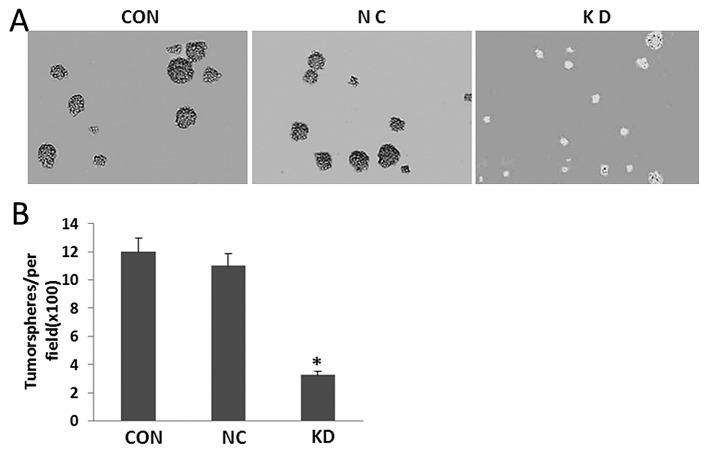
Knockdown of LGR5 leads to the inhibition of tumorsphere formation. (A) shRNA-LGR5/U87, shRNA-Ctr/U87 and non-transfected U87 cells (1,000) were plated for tumorsphere formation as described in Materials and methods which was quantified at ×100 magnification. (B) The number of tumorspheres in the shRNA-LGR5/U87 cells was significantly less than that in the shRNA-Ctr/U87 and non-transfected U87 cells (^*^P<0.05). CON, parental U87 cell; NC, negative control (shRNA-Ctr/U87); KD, LGR5 knockdown cells (shRNA-LGR5/U87).

To examine the self-renewal potential of U87 cells with or without LGR5 knockdown, we undertook tumorsphere formation culture of shRNA-LGR5/U87, shRNA-Ctr/U87 and untransfected U87 cells in a special ultra-low attachment culture plate with conditional medium for tumorsphere formation. After 7–10 days, plates were analyzed for tumorsphere formation which was quantified using an inverted microscope. As shown in Fig. 6, significantly fewer and smaller tumorspheres were observed in the shRNA-LGR5/U87 cells than those in the U87 and shRNA-Ctr/U87 cells (P<0.05; Fig. 6).

### LGR5 siRNA significantly inhibits tumorigenesis in vivo

**Figure 7:**
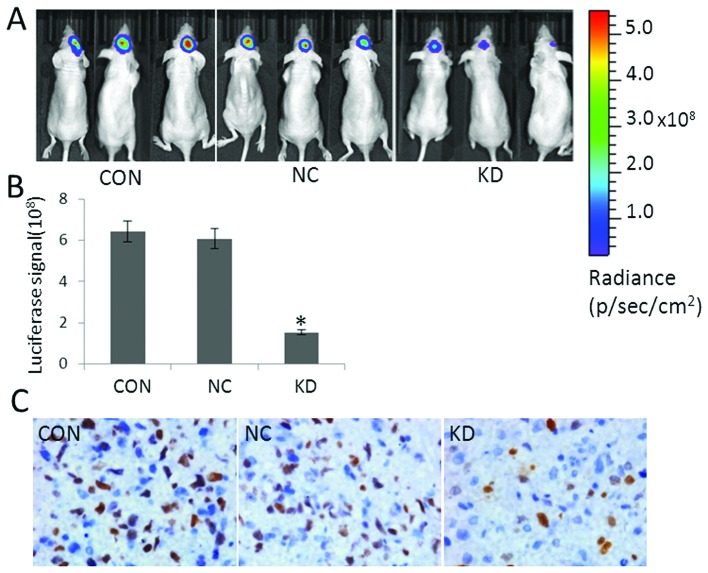
LGR5 depletion decreases tumorigenicity in nude mice. (A) Tumors were induced 5 weeks after the orthotopic implantation of control cells (U87 cell line), negative control cells and transfected U87 cells. Tumor growth in the mice was monitored by a live imaging system detecting the fluorescent signal. (B) The luciferase signal from shRNA-LGR5/U87 cells was significantly less than that from the shRNA-Ctr/U87 and non-transfected U87 cells (P<0.05). (C) Immunohistochemical analysis of LGR5 expression in tumors in nude mice 5 weeks following transplantation. CON, parental U87 cell; NC, negative control (shRNA-Ctr/U87); KD, LGR5 knockdown cells (shRNA-LGR5/U87).

To evaluate the effect of LGR5 on glioma *in vivo*, we injected shRNA-LGR5/U87, shRNA-Ctr/U87 and untransfected U87 cells into nude mice to develop orthotopic tumors. Tumor growth in the mice was monitored by a live imaging system detecting the fluorescent signals. During the course of the study, shRNA-LGR5/U87 cells demonstrated significantly inhibited tumor growth (Fig. 7A). All data were presented as the mean ± SD and as an average of three measurements from a representative experiment. The luciferase signal from shRNA-LGR5/U87 cells was significantly less than that from shRNA-Ctr/U87 and non-transfected U87 cells (P<0.05; Fig. 7B). Immunohistochemical analysis is shown in Fig. 7C)

## Discussion

LGR5 is a downstream target gene of the Wnt signaling pathway. The Wnt signaling pathway comprises a vast number of protein interactions and plays a critical role in tumorigenesis (6,21). Recently, studies have demonstrated the expression of LGR5 in several tumors including colorectal, gastric carcinoma, esophageal adenocarcinoma (8,9,15), liver (16), ovarian carcinoma (12) and Ewing sarcoma (22) and have shown that the overexpression of LGR5 is associated with poor prognosis. Our data demonstrated that the expression of LGR5 as detected by immunohistology was positively and markedly correlated with increasing WHO grade in the gliomas, which is consistent with the results of a previous report (17). It is well known that pathologic grade is linked to the degree of cell proliferation, and Ki-67 expression is associated with gene-regulated cellular growth and proliferation (23). We aimed to ascertain whether LGR5 expression is associated with the proliferation of glioma cells. The correlation between LGR5 expression and the proliferative index (PI) of tumor cells by Ki-67 staining was evaluated. Our results revealed that PI was positively related to LGR5 expression indicating LGR5 may be involved in cell proliferation in gliomas.

To confirm this hypothesis, we explored the role of LGR5 in three representative glioma cell lines. The data showed that LGR5 was highly expressed in all of the glioma cell lines at the protein and mRNA levels but the results did not achieve a significant difference. In addition, LGR5 expression was obviously decreased by RNA interference. U87 cells were randomly chosen for further examination. Downregulation of LGR5 resulted in suppression of cell proliferation, arrest of the cell cycle and reduction in clone formation. This evidence suggests a potential role for LGR5 in the regulation of glioma cell growth and proliferation which are in agreement with previous studies concerning basal cell carcinoma (13) and Ewing sarcoma (22). However, one recent report showed that suppression of LGR5 expression in colorectal cancer cells enhanced tumor formation with increased cell motility, while cells overexpressing LGR5 tended to grow with tight cell-to-cell contact and exhibited reduced cell motility (24). They considered that loss of LGR5 upregulates Wnt response genes and key EMT pathway genes. This paradoxical phenomenon warrants further study in order to investigate the gene function of LGR5 in different tissues and cells.

Recently, accumulating evidence supports the existence of glioma stem cells (GSCs), which have a high tumorigenic potential and are resistant to chemotherapy and irradiation (25,26). Researchers have suggested that GSCs might be responsible for tumor development and recurrence (27,28). As LGR5 is not only a target gene of the Wnt signaling pathway, but is also a stem cell marker in the intestine, stomach and hair follicles in the skin (5,29), we also detected the capability of tumorsphere formation by deregulating the expression of LGR5 in a glioma cell line. Consistent with a previous study (17), which showed that LGR5 knockdown suppresses viability and induces apoptosis of brain cancer stem cells, our data revealed that LGR5 knockdown inhibited tumorsphere formation. These results indicate that high levels of LGR5 expression may confer some of the properties of stem cells to tumor cells.

Additionally, to investigate the function of LGR5 in glioma *in vivo*, siRNA-transfected and parental U87 cells were orthotopically transplanted into nude mice. The results revealed that LGR5 depletion significantly suppressed tumor growth in nude mice. To the best of our knowledge, this is the first report showing the potent activity of LGR5 on glioma growth in an orthotopic xenograft model. Our result is consistent with a previous report which showed that LGR5-overexpressing HaCaT cells resulted in tumor formation when transplanted into nude mice (13). Moreover, Fukuma *et al*(16) reported that overexpression of LGR5 in hepatocellular carcinoma cells contributes to obvious nodular tumors. We deduce that, as LGR5 is a marker of GSCs (17), knockdown of LGR5 may resulted in the reduction of the capability of tumorigenesis *in vivo* which is the most prominent property of GSCs.

In the present study, we found that LGR5 transcriptional levels were upregulated in gliomas. The mechanisms by which oncogenic mutations and alterations in signaling pathways lead to the upregulation of LGR5 protein expression is an important issue concerning glioma cells. Recently, some researchers noted that the depletion of LGRs abrogated the synergistic effects of R-spondins on Wnt signaling, indicating that activation of the Wnt signaling pathway by R-spondins internalized together with LGR5 may contribute to the upregulation of LGR5 (5,30). However, the exact mechanisms behind this transcriptional upregulation of LGR5 and its promotion of proliferation in glioma remain to be illuminated.

In conclusion, our data demonstrated a correlation between LGR5 expression and the proliferation index in glioma, and knockdown of LGR5 resulted in suppression of glioma cell proliferation *in vitro* and *in vivo*. Therefore, LGR5 may be a valuable biomarker for the molecular diagnosis and a novel target for gene therapy of malignant gliomas.

## Figures and Tables

**Table I:** LGR5 IRS and PI in human gliomas of different pathologic grade.

Pathologic classification	n	LGR5 IRS	PI (%)
Grade I	5	2.58±0.30	11.90±2.28
Grade II	13	3.52±0.87	26.15±4.22
Grade III	13	5.14±1.47	36.44±13.5
Grade IV	23	7.89±1.84	36.44±13.5
Total	54	4.60±2.57	47.11±10.1
P-value^a^		<0.01	<0.01
P-value^b^		<0.01	<0.01

a Difference in LGR5 IRS and PI in gliomas categorized in different pathologic grade groups was compared using analysis of variance (ANOVA).

b The Spearman rank test was used to establish the correlation between pathologic grade and LGR5 IRS and PI. Values are expressed as mean ± SD.
